# Augmenting healthy brain magnetic resonance images using generative adversarial networks

**DOI:** 10.7717/peerj-cs.1318

**Published:** 2023-04-11

**Authors:** Sarah S. Alrumiah, Norah Alrebdi, Dina M. Ibrahim

**Affiliations:** 1 Department of Information Technology, College of Computer, Qassim University, Buraydah, Saudi Arabia; 2 Department of Computers and Control Engineering, Faculty of Engineering, Tanta University, Tanta, Egypt

**Keywords:** Brain tumors magnetic resonance imagings (MRIs), Generative adversarial networks (GANs), Image augmentation

## Abstract

Machine learning applications in the medical sector face a lack of medical data due to privacy issues. For instance, brain tumor image-based classification suffers from the lack of brain images. The lack of such images produces some classification problems, *i.e.*, class imbalance issues which can cause a bias toward one class over the others. This study aims to solve the imbalance problem of the “no tumor” class in the publicly available brain magnetic resonance imaging (MRI) dataset. Generative adversarial network (GAN)-based augmentation techniques were used to solve the imbalance classification problem. Specifically, deep convolutional GAN (DCGAN) and single GAN (SinGAN). Moreover, the traditional-based augmentation techniques were implemented using the rotation method. Thus, several VGG16 classification experiments were conducted, including (i) the original dataset, (ii) the DCGAN-based dataset, (iii) the SinGAN-based dataset, (iv) a combination of the DCGAN and SinGAN dataset, and (v) the rotation-based dataset. However, the results show that the original dataset achieved the highest accuracy, 73%. Additionally, SinGAN outperformed DCGAN by a significant margin of 4%. In contrast, experimenting with the non-augmented original dataset resulted in the highest classification loss value, which explains the effect of the imbalance issue. These results provide a general view of the effect of different image augmentation techniques on enlarging the healthy brain dataset.

## Introduction

Medical images are highly used in the technical field to automate several tasks. Automation helps enhance the repetitive tasks related to detecting diseases, finding relations between diseases, *etc.* Moreover, automation of repetitive tasks helps to reduce time and cost besides, assists in early disease detection. Early detection of the diseases helps patients’ response to treatment and prevents the exacerbation of the disease and death in some cases.

The brain tumor is a cancer type considered a common cause of death globally (Elazab et al., 2020). Thus, brain tumors have a critical need for early detection. A brain tumor is an abnormal growth of brain cells that seriously affects the central nervous system (Arbane et al., 2021). Researchers and developers work on training machine learning models to identify abnormal cases. However, due to the patients’ privacy, there is a lack of available medical images, including brain tumor images. The lack of datasets causes an imbalanced classification problem. Imbalance classification occurs when the model trains with unequal distribution of classes (Thabtah et al., 2020).

Several studies resorted to augmentation methods to generate more data. Hence, some studies used traditional augmentation techniques to generate medical images (Naseer et al., 2021). However, conventional techniques may not be suitable for all types of images. Thus, advanced augmentation methods have been developed, such as generative adversarial networks. A generative adversarial network (GAN) is a neural network that contains two networks: (i) generator network and (ii) discriminator network (Yi, Walia & Babyn, 2019). Both networks work simultaneously, the generator generates fake images while the discriminator discriminates between real and fake photos. GAN techniques were used to enlarge the medical images’ datasets, *e.g.*, augmenting brain tumor images. Moreover, GAN was used to automate the image segmentation process (Wu et al., 2021) and augment brain tumor images (Ge et al., 2020). However, most of the efforts focused on generating magnetic resonance images (MRIs) identifying tumors. Thus, to the best of the authors’ knowledge, limited studies discussed the effect of augmenting healthy brain MRIs on brain tumor classification performance.

The lack of studies discussing the class imbalance issue that occurs in classifying brain MRIs motivated the authors to use augmentation techniques to solve the imbalance issue. This study aims to augment healthy brain MRIs using GAN-based augmentation techniques to solve the imbalanced classification problem in brain tumors that happen due to the lack of healthy brain images. The imbalanced classification problem appears when the used dataset contains an imbalanced number of data in each class, *e.g.*, 60% of the data are class A while the remaining 40% are class B data. In this case, the model trains on class A data more than other classes, which results in a model bias toward the majority class (class A in our example). Thus, most of the data might be incorrectly classified as class A data. Therefore, the study’s contributions are:

• Augment “no tumor” brain MRIs in Sartaj et al. (2019) using two different GANs (i) multi-input augmentation technique (deep convolutional GAN (DCGAN)) and (ii) single-input augmentation technique (single GAN (SinGAN)) to avoid imbalance classification.
• Study the effect of applying GAN-based techniques to generate healthy brain MRIs.
• Classify different versions of brain MRI datasets using the VGG16 classifier (Simonyan & Zisserman, 2015).

The rest of the paper is structured as follows; Section 2 discusses the GAN-based related work on brain tumor MRIs. Section 3 describes the followed methodology. Section 4 presents the experiments’ and results’ details. Section 5 discusses the outcomes and Section 6 concludes the study.

## Related Work

Various classification models have been developed to classify brain MRIs to detect if the MR scan represents a tumor or not. Those classification methods recorded reasonable performance with some classification issues related to the limited data available. Thus, image augmentation was proposed to solve such issues. Image augmentation can be performed using the classic or the intelligent approaches (Gab Allah, Sarhan & Elshennawy, 2021). The classic image augmentation techniques increase the dataset by using the existing data to generate new data using basic manipulations, such as geometric transformation (Shorten & Khoshgoftaar, 2019), whereas the intelligent image augmentation techniques, such as GAN-based methods, enlarge the dataset by generating new data that differ from the existing data.

According to brain tumor GAN-based studies, the usage of GANs is divided into two main applications: (i) image augmentation and (ii) image segmentation. However, this section will focus on the image augmentation techniques used to enlarge brain tumor MRIs to solve the class imbalance issue.

Several studies used GAN-based models to generate new medical images (Qin et al., 2020; Chi et al., 2018). Brain tumor images are one of the medical images used in several image augmentation studies. For instance,  Ge et al. (2020) implemented a pairwise GAN model on brain MRIs. Moreover, they classified the glioma subtype using a slice-level and then used a majority voting to identify the diagnostic on the patient level. Another GAN method was proposed in Kim, Kim & Park (2021) that used a normal brain image and an image of a simplified tumor mask in a circle shape. The generator converted the circles into different tumor masks and then painted them on the authentic normal brain images generating an image similar to the real images.

Additionally, Sandhiya et al. (2021) used DCGAN to generate three kinds of malignant tumors: meningioma, glioma, and pituitary to enhance the classification process. In contrast, Biswas et al. (2021) augmented tumor images using Wasserstein GANs (WGANs) to improve the segmentation of the image. Moreover, Han et al. (2020) proposed a multi-stage progressive growth GAN (PGGA) to generate new images combined with a traditional data augmentation technique such as geometric transformations. The integration in Han et al. (2020) enhanced the classification performance compared with images generated using PGGAN only. Similarly, PGGANs were used in Han et al. (2019), where the authors used two-step GAN-based data augmentation. In the first step, PGGANs generated high-resolution images. In contrast, the second step applied noise-to-image and image-to-image GANs to enhance the data augmentation effect with the GAN techniques. Table 1 summarizes the brain tumor augmentation-based studies.

**Table 1:** Brain tumor augmentation related studies.

Ref	Year	GAN type	Dataset	Number of images	Partitioned dataset	Classification	Experiments	Results
Ge et al. (2020)	2020	pairwise GANs	3D brain images from TCGA-GBM and TCGA-LGG (Glioma Types)	-Original Dataset = 1002 -Augmented Dataset = 1782	Training = 86% Validation = 7% Testing = 7%	multi-stream 2D CNN, feature-level fusion	Original + GAN-based DA	Accuracy = 88.82% Sensitivity = 81.81% Specificity = 92.17%
Kim, Kim & Park (2021)	2021	Three G and one D approach	BraTS 2018	Original Dataset = 12540	Training = 12500	U-NET	-Original -Traditional DA -GAN-based DA	GAN-based DA: FID = 1.16 Dice = 59% Sensitivity = 54% Precision = 70%
Sandhiya et al. (2021)	2021	DCGAN	4 Labels: -Glioma -Meningioma -Pituitary -No tumors	-Original Dataset = 1000 -Augmented Dataset = 2920	Training = 80% Validation = 13% Testing = 7%	Faster R-CNN	Original + GAN-based DA	Accuracy = 89.8% F1-score = 0.89
Biswas et al. (2021)	2021	WGAN	BRATS 2013	–	–	Random Forest	Original + GAN-based DA	DSC = 94% JSC = 89%
Han et al. (2020)	2020	PGGAN + Wasserstein loss	BraTS 2016	Original Dataset = 12979	Training = 69% Validation = 11% Testing = 20%	ResNet-50	-Original dataset -Traditional DA -GAN-based DA -Traditional DA + GAN-based DA	200k traditional DA+200k PGGAN-based DA: Accuracy = 91.08% Specificity = 97.6%
Han et al. (2019)	2019	PGGANs + MUNIT or SimGAN	BraTS 2016	-Original Dataset = 8,429 -Augmented Dataset = 200k, 400k	Training = 70% Validation = 11% Testing = 19%	ResNet-50	-Original dataset -Traditional DA - GAN-based DA -Traditional DA + GAN-based DA	-(+) 200k traditional DA & 200k MUNIT-refined DA: Accuracy = 96.7 Sensitivity = 95.45

Even though the deep learning-based emerging image augmentation techniques reported good performance, researchers still tend to apply classic image augmentation methods to increase their training set (Cirillo, Abramian & Eklund, 2021; Ghassemi, Shoeibi & Rouhani, 2020). Moreover, major studies focused on glioma tumors regarding the availability of glioma-based BraTS datasets (Wu et al., 2021; Chen et al., 2019; Li, Chen & Shi, 2021; Alex et al., 2017; Cirillo, Abramian & Eklund, 2021; Nema et al., 2020).

According to the reviewed studies, the generated GAN-based images were used only in the training set to train the model. Moreover, it is worth noting that the synthetic GAN-based images improved the performance of the brain tumor classification in some cases (Ge et al., 2020; Biswas et al., 2021). On the other hand, the classic augmentation techniques improved the classification accuracy when combined with the GAN-based fake images in some studies (Han et al., 2020; Han et al., 2019). Thus, these results encouraged the authors to generate synthetic GANs-based and geometric argumentation-based images in the training part to solve the imbalance problem in the brain tumor MRI dataset and enhance the classification performance.

## Materials and Methods

**Figure 1:**
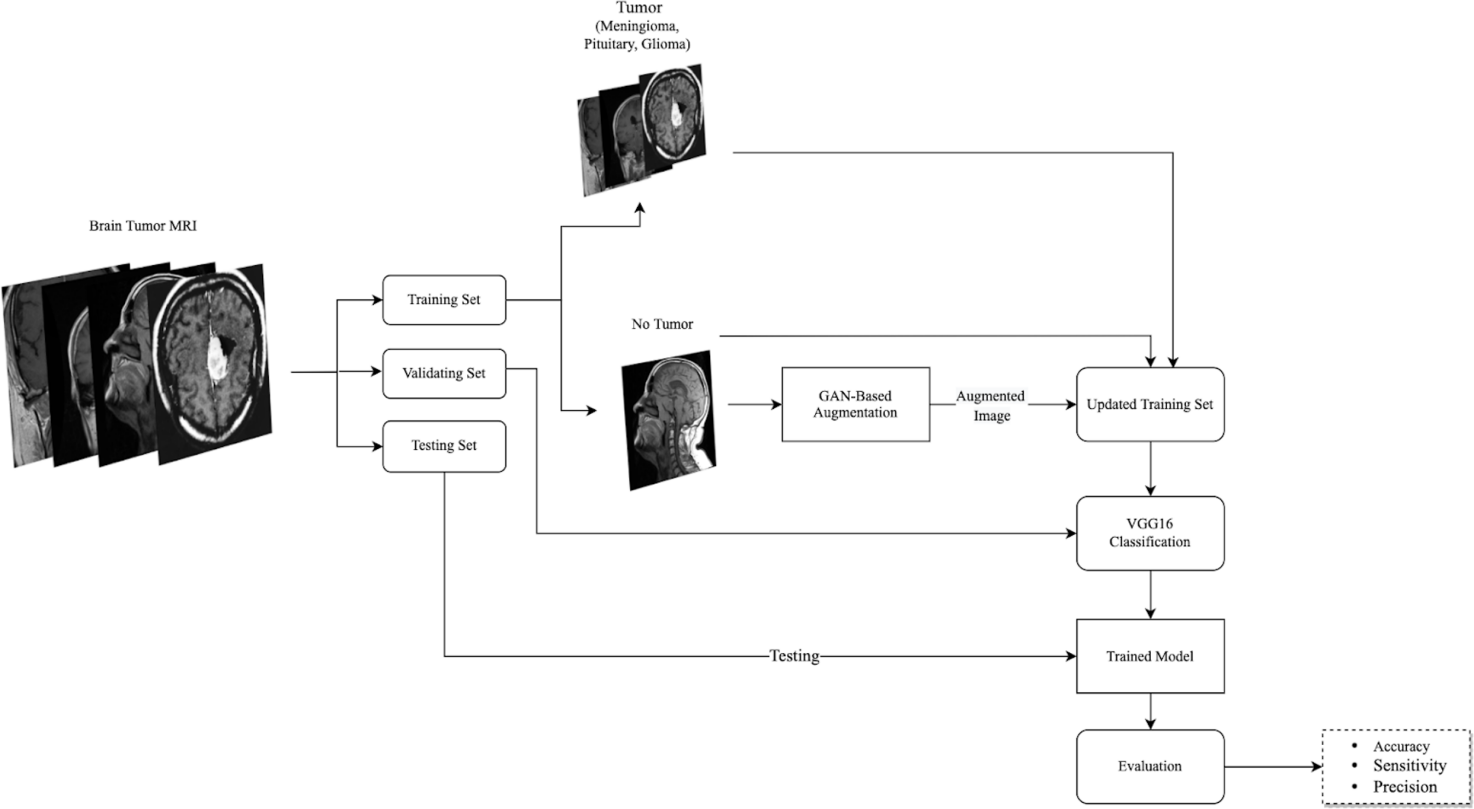
The followed methodology of augmenting and classifying brain tumor MRIs is described here. The used dataset consisted of four classes, three tumor types, and healthy MRIs. After splitting the dataset into training, validation, and testing sets, the no tumor images in the training set were augmented using GAN-based augmentation techniques and added to the training set. Then, a VGG16 classifier was trained and evaluated. (Brain images source: Sartaj et al., 2019).

This effort is dedicated to study the effect of brain MRIs augmentation on the classification performance using GAN-based augmentation techniques, *i.e.,* DCGAN and SinGAN. DCGAN has been used in augmenting brain MRIs and proved its effectiveness (Sandhiya et al., 2021). However, DCGAN generates images from a set of input images. Thus, the authors were encouraged to study the effect of using the single-input augmentation technique (SinGAN) and multi-input augmentation technique (DCGAN) on brain MRIs. Moreover, this work explores the effect of traditional augmentation techniques. Specifically, the rotation method was selected since it does not perform any changes in the internal details of the image. Therefore, the following subsections discuss the methodology phases’ details as illustrated in Fig. 1.

### Dataset

The authors used the brain MRIs dataset in Sartaj et al. (2019). The dataset contains MR images of three types of brain tumors (glioma, meningioma, and pituitary) and normal brain (no tumor) as well. The dataset consists of 3264 RGB JPG images. Table 2 presents the dataset contents. Based on Table 2, the dataset suffers from two issues, (i) class imbalance and (ii) random splitting ratios. The number of “no tumor” images is relatively small compared with the tumor ones, which is 500, 937, 901, and 926 images of no tumor, meningioma, pituitary, and glioma tumors, respectively. Thus, this difference causes imbalance classification issues where the classifier might bias toward tumors’ scans. As a solution, the no tumor images can be augmented using GAN techniques. Moreover, the train-test splitting ratio of the “Pituitary Tumor” images is unfair compared with the rest. Therefore, the dataset needs to be re-split with appropriate ratios.

### Image augmentation

**Figure 2:**
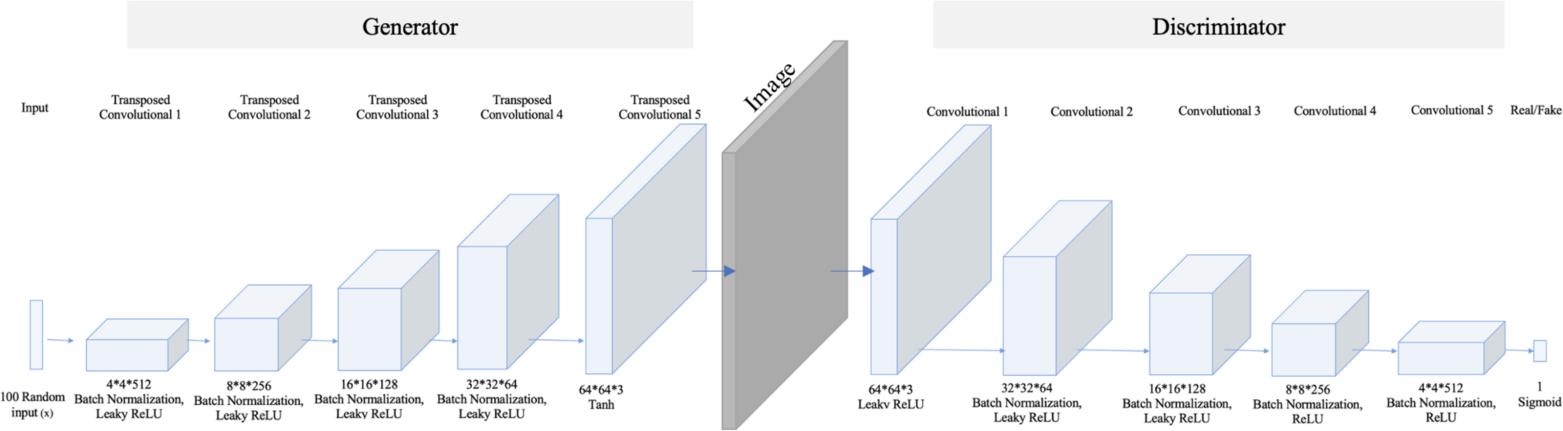
DCGAN architecture. The model comprises five transposed convolution layers in the generator and five convolution layers in the discriminator. Each layer in the generator used Rectified Linear Unit (ReLu) as an activation method except the last layer, which used a hyperbolic tangent (Tanh) function. While in the discriminator, Leaky ReLu is used as an activation method in all hidden layers and sigmoid in the last layer.

Regarding the small number of “no tumor” images, two GAN-based augmentation techniques have been applied on the “no tumor” images to increase them. DCGAN and SingleGAN were used. Additionally, the geometric-based traditional augmentation method has been experimented with using the rotation method with 90 and 270 degrees. DCGAN is a deep convolutional GAN architecture that replaces the pooling layers in the original GAN with strided convolutions in the discriminator and fractional-strided convolutions in the generator (Radford, Metz & Chintala, 2016). DCGAN uses batch normalization and does not include fully connected hidden layers. Figure 2 illustrates the DCGAN architecture and specifies the activation functions used in the generator and discriminator.

**Table 2:** Brain MRIs dataset contents.

Image label	Training set size	Testing set size	Total
Meningioma Tumor	822 (88%)	115 (12%)	937 (28%)
Pituitary Tumor	**827 (92%)**	**74 (8%)**	901 (28%)
Glioma Tumor	826 (89%)	100 (11%)	926 (28%)
No Tumor	395 (79%)	105 (21%)	**500 (15%)**
Total	2870 (88%)	394 (12%)	3264 (100%)

**Notes.**

The bold text indicates the random splitting ratios and imbalance issues.

**Figure 3:**
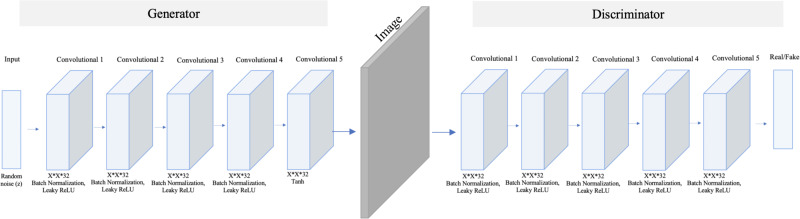
SinGAN architecture. The model comprises five convolution layers in both the generator and discriminator. Moreover, each layer used Leaky ReLu as an activation method except the last layer in the generator, which used a Tanh function.

In contrast, SinGAN is an unconditional GAN model that learns from a single image (Shaham, Dekel & Michaeli, 2019). Therefore, SinGAN successfully generates several fake images by capturing the patches of internal distribution in the real image. Specifically, SinGAN consists of a pyramid of fully convolutional GANs, and each of them learns the patch of internal distribution of the real image in different scales. Furthermore, SinGAN uses a 3*3 kernel size, and it generates images with a size equal to the input image size. Thus, this structure enables SinGAN to generate diverse fake images that maintain the main structure of the real image. Figure 3 presents the SinGAN architecture at scale 0.

**Figure 4:**
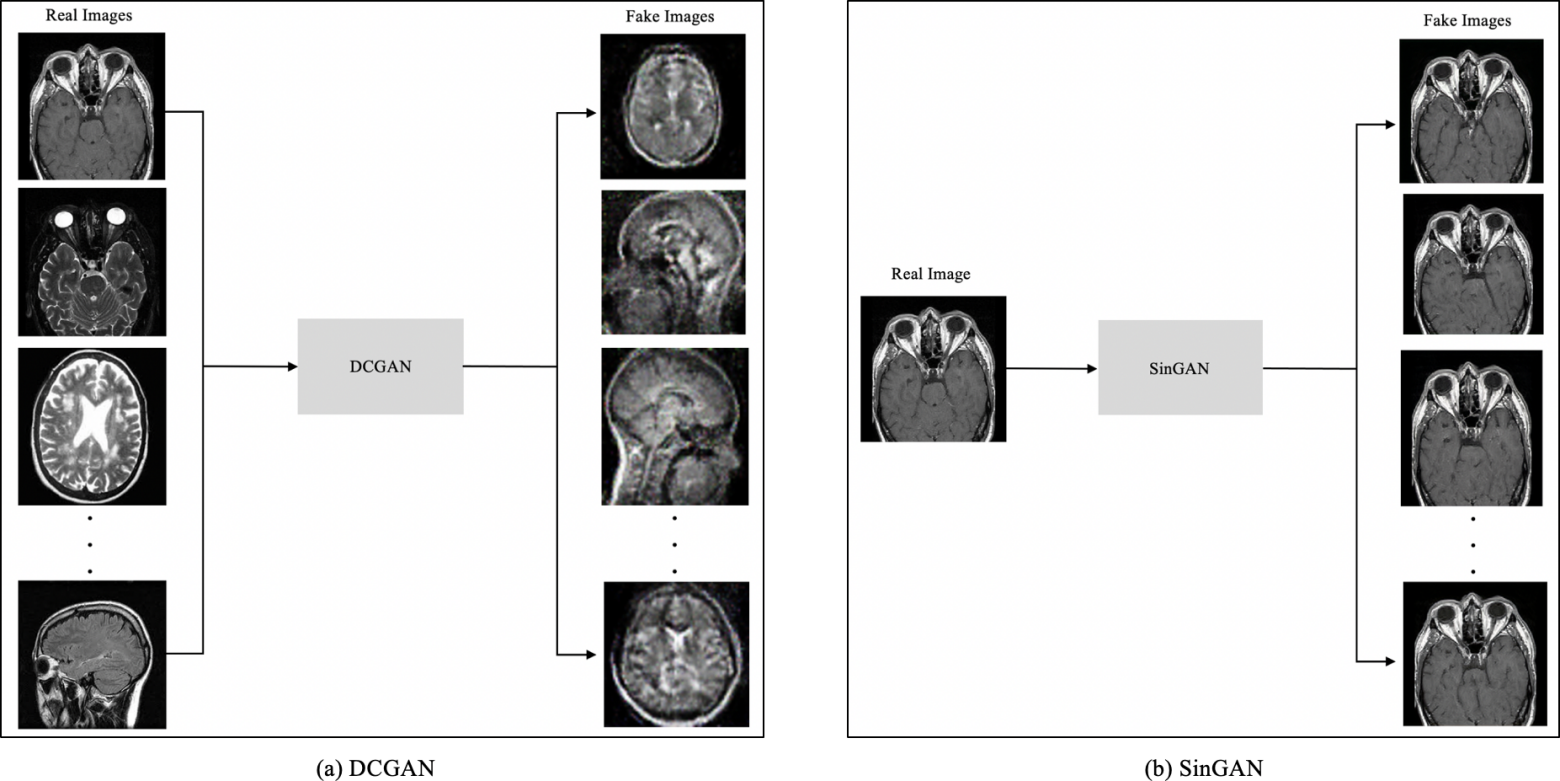
The input size difference between DCGAN and SinGAN. (Brain images source: Sartaj et al., 2019).

The main difference between DCGAN and SinGAN is in the input size. DCGAN accepts multiple input images to produce a set of fake images. While SinGAN accepts a single input image to produce a set of fake images (see Fig. 4).

### Image classification

**Figure 5:**
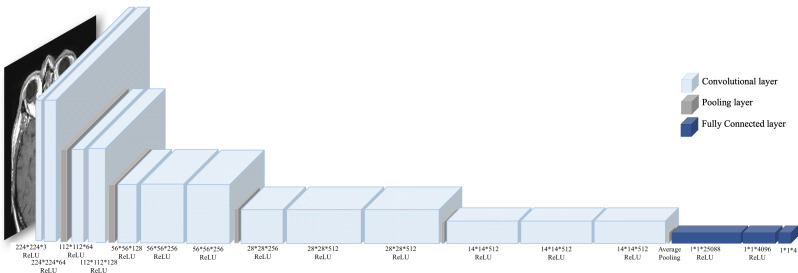
VGG16 architecture. VGG16 consists of 13 convolutional layers with ReLU activation function, three fully connected layers, and five pooling layers. (Brain image source: Sartaj et al., 2019).

As mentioned before, the brain tumor MRI dataset contains four classes: (i) glioma tumor, (ii) meningioma tumor, (iii) pituitary tumor, and (iv) no tumor. Therefore, the pre-trained VGG16 classifier was used to classify brain tumor MRIs. VGG16 is a deep convolutional neural network (CNN) architecture designed to win the ImageNet challenge in 2014 (Simonyan & Zisserman, 2015). VGG16 increases the depth of the CNN using 3*3 convolution filters and 16 deep layers. Additionally, VGG16 has been trained on large datasets, *i.e.,* millions of images; thus, it learned many features. Figure 5 presents the architecture of VGG16.

### Evaluation

The authors conducted a review of evaluation criteria used in previous studies to determine the evaluation criteria for this study. Table 3 shows that the confusion matrix values were mostly used besides F1-score, Frechet Inception Distance (FID), Dice Similarity Coefficient (DSC), and Jaccard Similarity Coefficient (JSC). FID measure was used in this study to measure the quality of the GAN-based generated images. FID calculates the similarity score between two sets of images, the GAN-based generated images and the original images (Heusel et al., 2017). A lower FID score indicates better image quality and low noise. Moreover, in terms of evaluating the classification performance, this study followed the previous studies’ approach by using several measures extracted from the confusion matrix. In particular, (i) accuracy, (ii) sensitivity (recall), and (iii) precision were selected as the main evaluation criteria in this study.

**Table 3:** Evaluation criteria of classification performance in augmentation-related studies.

Ref	Accuracy	Sensitivity	Specificity	Precision	F1-Score	FID	DSC	JSC
Elazab et al. (2020)	✓	✓	✓	–	–	–	–	-
Thabtah et al. (2020)	–	✓	–	✓	–	✓	✓	-
Yi, Walia & Babyn (2019)	✓	–	–	–	✓	–	–	-
Sartaj et al. (2019)	–	–	–	–	–	–	✓	✓
Qin et al. (2020)	✓	✓	✓	–	–	–	–	-
Chi et al. (2018)	✓	✓	✓	–	–	–	–	-
Ours	✓	✓	–	✓	–	–	–	-

To calculate each measure, several values are used as explained in Ting (2017), which are illustrated as follows:

**True Positive (TP):** the image is a tumor, and the classifier is classified correctly.

**False Positive (FP):** the image is no tumor, and the classifier is classified incorrectly.

**True Negative (TN):** the image is no tumor, and the classifier is classified correctly.

**False Negative (FN):** the image is a tumor, and the classifier is classified incorrectly.

Thus, accuracy, sensitivity (recall), and precision are introduced as follows, respectively:

\begin{eqnarray*}Accuracy= \frac{TP+TN}{TP+FP+TN+FN} \end{eqnarray*}

\begin{eqnarray*}Sensitivity= \frac{TP}{TP+FN} \end{eqnarray*}

\begin{eqnarray*}Precision= \frac{TP}{TP+FP} . \end{eqnarray*}

## Experiments and Results

This section discusses the results of the augmentation and classification steps.

### Image augmentation

After conducting many augmentation experiments and fine-tuning the parameters to get reasonable results, Table 4 illustrates the parameters used to augment “no tumor” brain images. Notably, these experiments aim to get the best result for each technique separately, not to standardize the parameters, which explains the difference between the techniques’ parameter values (number of epochs and learning rates) illustrated in Table 4. Regarding augmenting with DCGAN, “no tumor” images were resized to 64 and normalized. Images were normalized to be in the range of −1 to 1 using 0.5 mean and 0.5 standard deviation. After many experiments using 128 and 64 batch sizes, the authors noticed that the DCGAN performance improved when the batch size decreased, *i.e.,* 64. Tables 5 and 6 illustrate the generator and discriminator models in both DCGAN and SinGAN models.

**Figure 6:**
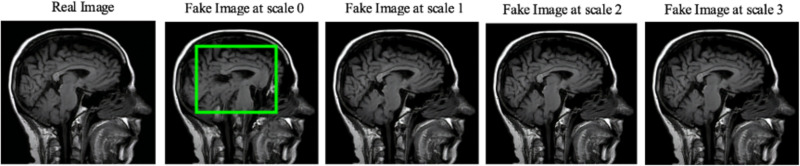
SinGAN generated images at different scales. (Brain images source: Sartaj et al., 2019).

**Figure 7:**
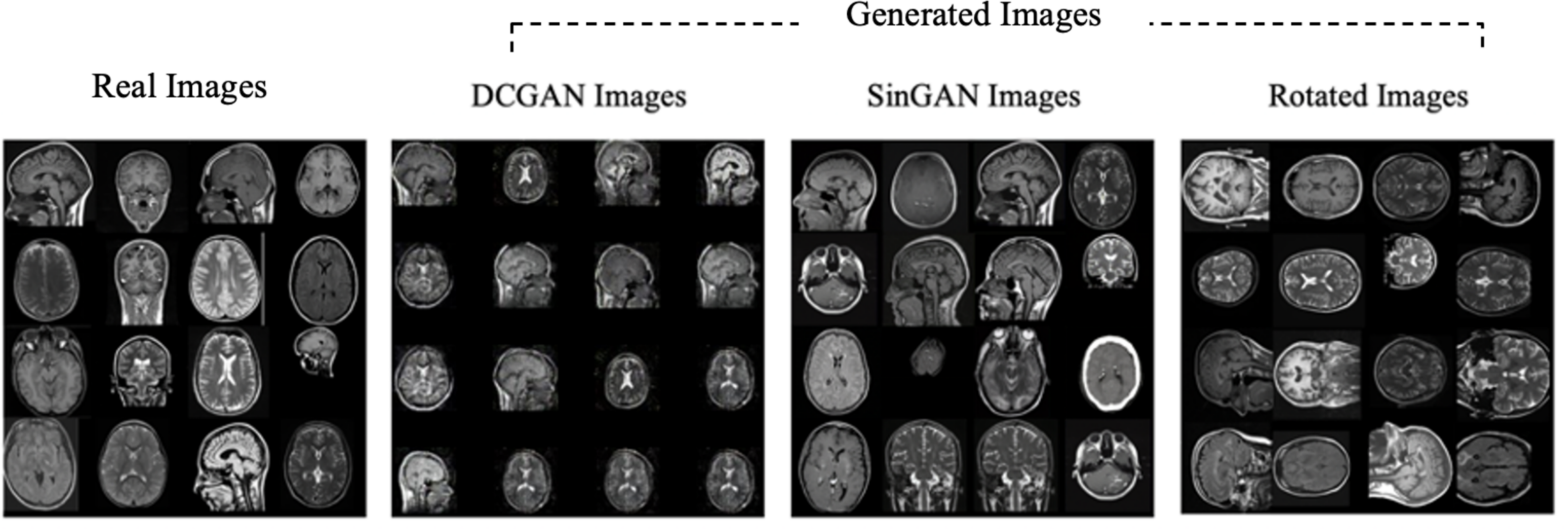
Real “no tumor” images compared with DCGAN, SinGAN, and geometric-based generated images. (Brain images source: Sartaj et al., 2019).

Moreover, the “no tumor” dataset contains images from different angles, *e.g.*, the side of the head or top. Therefore, the DCGAN performance differed when it was trained with (i) all the images, (ii) images taken from the side, and (iii) images taken from the top angle (see Table 7). The performance of the DCGAN generator and discriminator was enhanced when the images were unified, *i.e.,* side-only images and top-only images. On the other hand, SinGAN generates a fake image at different scales related to how the generated image is realistic. Usually, the scales range from 0–3, where 0 will generate a completely different image from the original one. In contrast, other scales will generate a new image with minor changes from the original image. Any scale number above three will generate a new image almost identical to the original one. Figure 6 shows the difference between the generated images at each scale. As it appears, the images at scales 1, 2, and 3 are similar. Moreover, these images are similar to the real image, unlike the zero-scale image, which has noticeable changes. All the training images generated in this study were at a scale of 1 to avoid generating completely different or identical images to the original ones. Furthermore, Fig. 7 compares the fake “no tumor” images generated by DCGAN and SinGAN with the real images. Additionally, the FID score was reported in Table 8 to measure the similarities between the “no tumor” DCGAN and SinGAN generated images compared to the original “no tumor” images. A lower FID score determines the high similarity between the original and generated images. Therefore, from Fig. 7 and Table 8, it is noticeable that SinGAN images were more similar to the real “no tumor” images.

**Table 4:** Augmentation parameters.

Augmentation technique	Epochs	Learning rate	Batch size	Generated image size	Noise size	Loss function	Optimizer
DCGAN	6000	0.0002	64	64	100	Binary cross entropy	Adam
SinGAN	7000	0.0005	–	same size of the input image	–	Wasserstein GAN- gradient penalty	Adam

**Table 5:** DCGAN generator and discriminator networks details.

Generator		
Layer (type)	Output shape	Param #
ConvTranspose2d-1	[None, 512, 67, 67]	819,200
BatchNorm2d-2	[None, 512, 67, 67]	1,024
ReLU-3	[None, 512, 67, 67]	0
ConvTranspose2d-4	[None, 256, 134, 134]	2,097,152
BatchNorm2d-5	[None, 256, 134, 134]	512
ReLU-6	[None, 256, 134, 134]	0
ConvTranspose2d-7	[None, 128, 268, 268]	524,288
BatchNorm2d-8	[None, 128, 268, 268]	256
ReLU-9	[None, 128, 268, 268]	0
ConvTranspose2d-10	[None, 64, 536, 536]	131,072
BatchNorm2d-11	[None, 64, 536, 536]	128
ReLU-12	[None, 64, 536, 536]	0
ConvTranspose2d-13	[None, 3, 1072, 1072]	3,072
Tanh-14	[None, 3, 1072, 1072]	0
Total params: 3,576,704		
Trainable params: 3,576,704		
Non-trainable params: 0		
Discriminator		
Conv2d-1	[None, 64, 32, 32]	3,072
LeakyReLU-2	[None, 64, 32, 32]	0
Conv2d-3	[None, 128, 16, 16]	131,072
BatchNorm2d-4	[None, 128, 16, 16]	256
LeakyReLU-5	[None, 128, 16, 16]	0
Conv2d-6	[None, 256, 8, 8]	524,288
BatchNorm2d-7	[None, 256, 8, 8]	512
LeakyReLU-8	[None, 256, 8, 8]	0
Conv2d-9	[None, 512, 4, 4]	2,097,152
BatchNorm2d-10	[None, 512, 4, 4]	1,024
LeakyReLU-11	[None, 512, 4, 4]	0
Conv2d-12	[None, 1, 1, 1]	8,192
Sigmoid-13	[None, 1, 1, 1]	0
Total params: 2,765,568		
Trainable params: 2,765,568		
Non-trainable params: 0		

**Table 6:** SinGAN generator networks details at scale 0.

Generator		
Layer (type)	Output shape	Param #
Conv2d-1	[None, 32, 98, 98 ]	896
BatchNorm2d-2	[None, 32, 98, 98 ]	64
LeakyReLU-3	[None, 32, 98, 98 ]	0
Conv2d-4	[None, 32, 98, 98 ]	896
BatchNorm2d-5	[None, 32, 98, 98 ]	64
LeakyReLU-6	[None, 32, 98, 98 ]	0
Conv2d-7	[None, 32, 98, 98 ]	896
BatchNorm2d-8	[None, 32, 98, 98 ]	64
LeakyReLU-9	[None, 32, 98, 98 ]	0
Conv2d-10	[None, 32, 98, 98 ]	896
BatchNorm2d-11	[None, 32, 98, 98 ]	64
LeakyReLU-12	[None, 32, 98, 98 ]	0
Conv2d-13	[None, 32, 98, 98 ]	896
Tanh-14	[None, 3, 98, 98 ]	0
Total params: 4736		
Discriminator		
Conv2d-1	[None, 32, 98, 98]	896
BatchNorm2d-2	[None, 32, 98, 98]	64
LeakyReLU-3	[None, 32, 98, 98]	0
Conv2d-4	[None, 32, 98, 98 ]	896
BatchNorm2d-5	[None, 32, 98, 98]	64
LeakyReLU-6	[None, 32, 98, 98 ]	0
Conv2d-7	[None, 32, 98, 98 ]	896
BatchNorm2d-8	[None, 32, 98, 98 ]	64
LeakyReLU-9	[None, 32, 98, 98 ]	0
Conv2d-10	[None, 32, 98, 98]	896
BatchNorm2d-11	[None, 32, 98, 98 ]	64
LeakyReLU-12	[None, 32, 98, 98 ]	0
Conv2d-13	[None, 1, 1, 1]	896
Total params: 4736		

**Table 7:** DCGAN generator and discriminator performance trained in 6000 epochs. (Brain images source: Sartaj et al., 2019).

Type of images	All no tumor MRIs	No tumor MRIs taken from the side	No tumor MRIs taken from the top side only
Size	395	91	251
Generator and discriminator loss	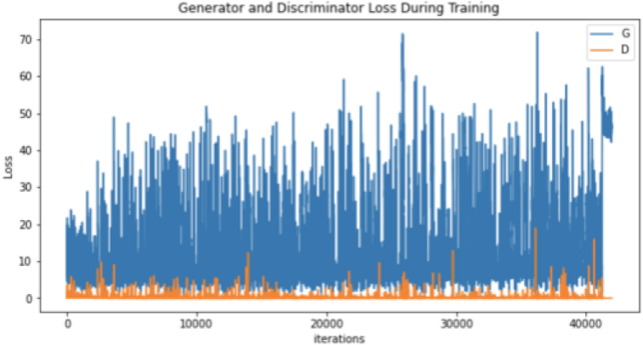	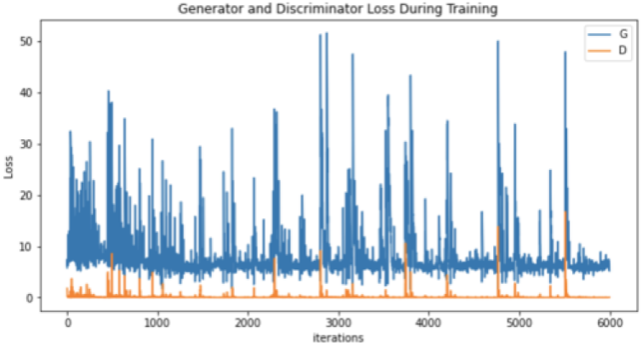	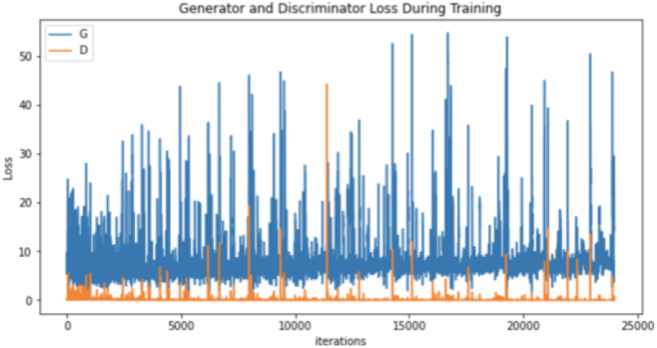
Sample of generated images	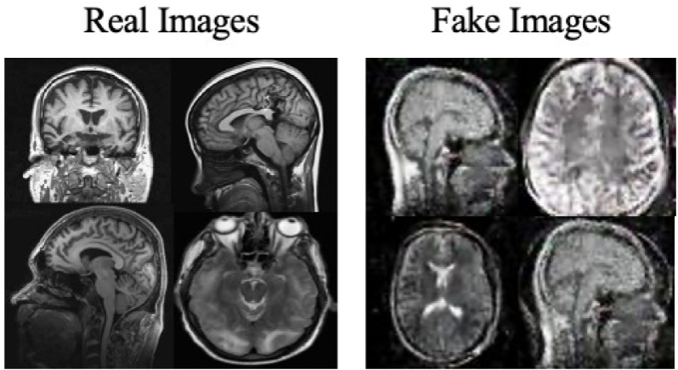	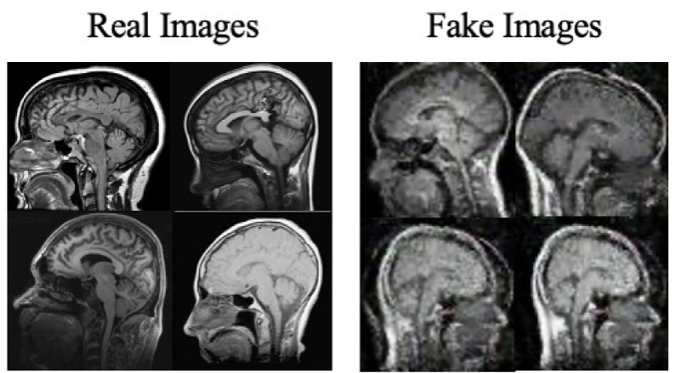	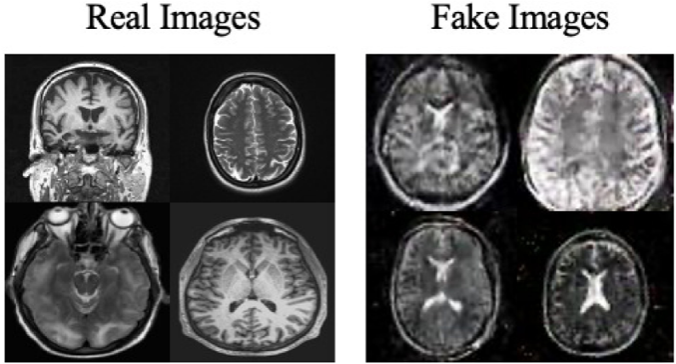

### Image classification

**Figure 8:**
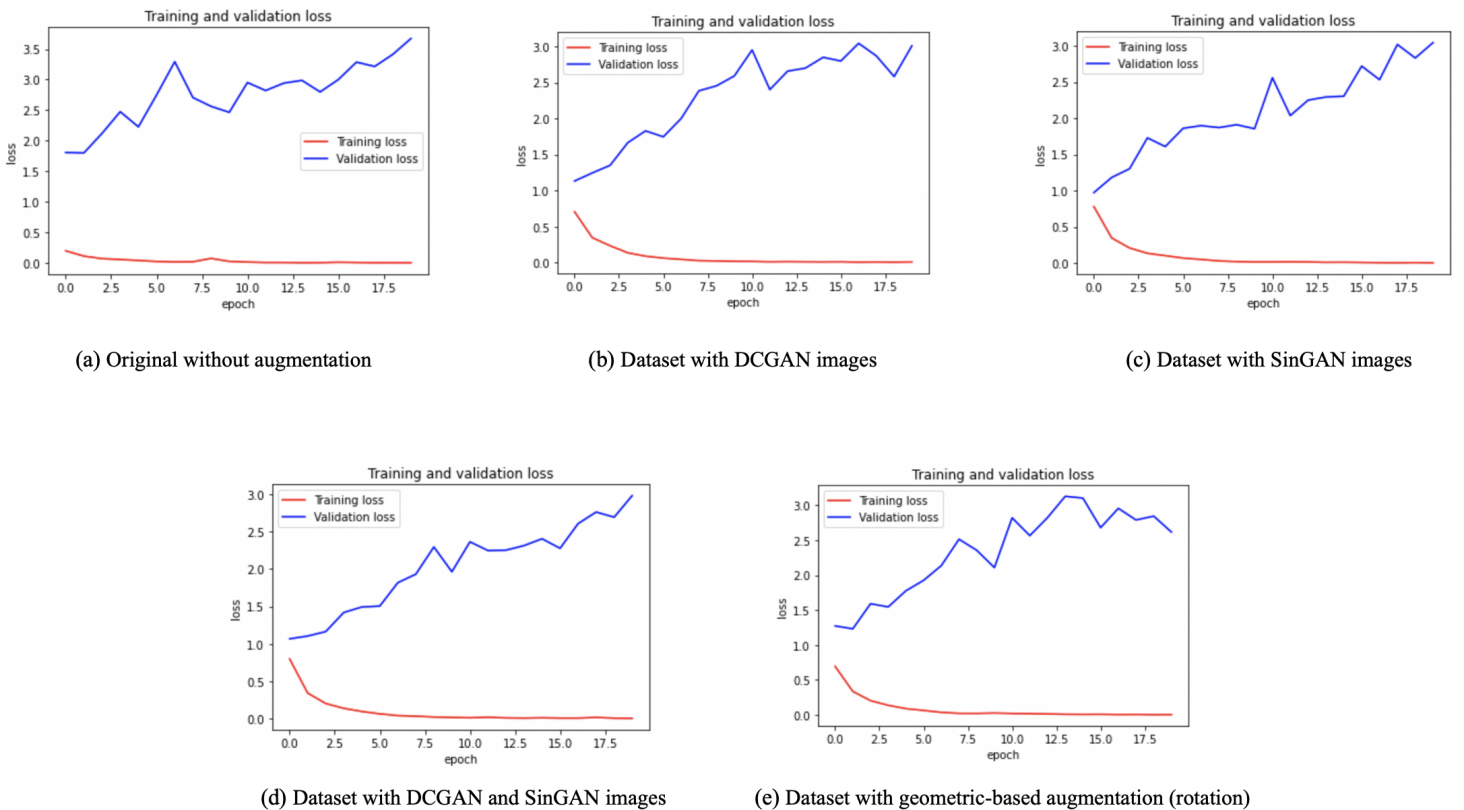
Training and validation loss for each dataset. (A) The overfitting problem in the classifier performance with the original imbalanced dataset. (B, C, D) The training and validation loss after augmenting the dataset using GAN-based techniques. (E) The loss reported with the geometric-based augmented dataset. The gap between training and validation loss was reduced with the augmented datasets.

**Figure 9:**
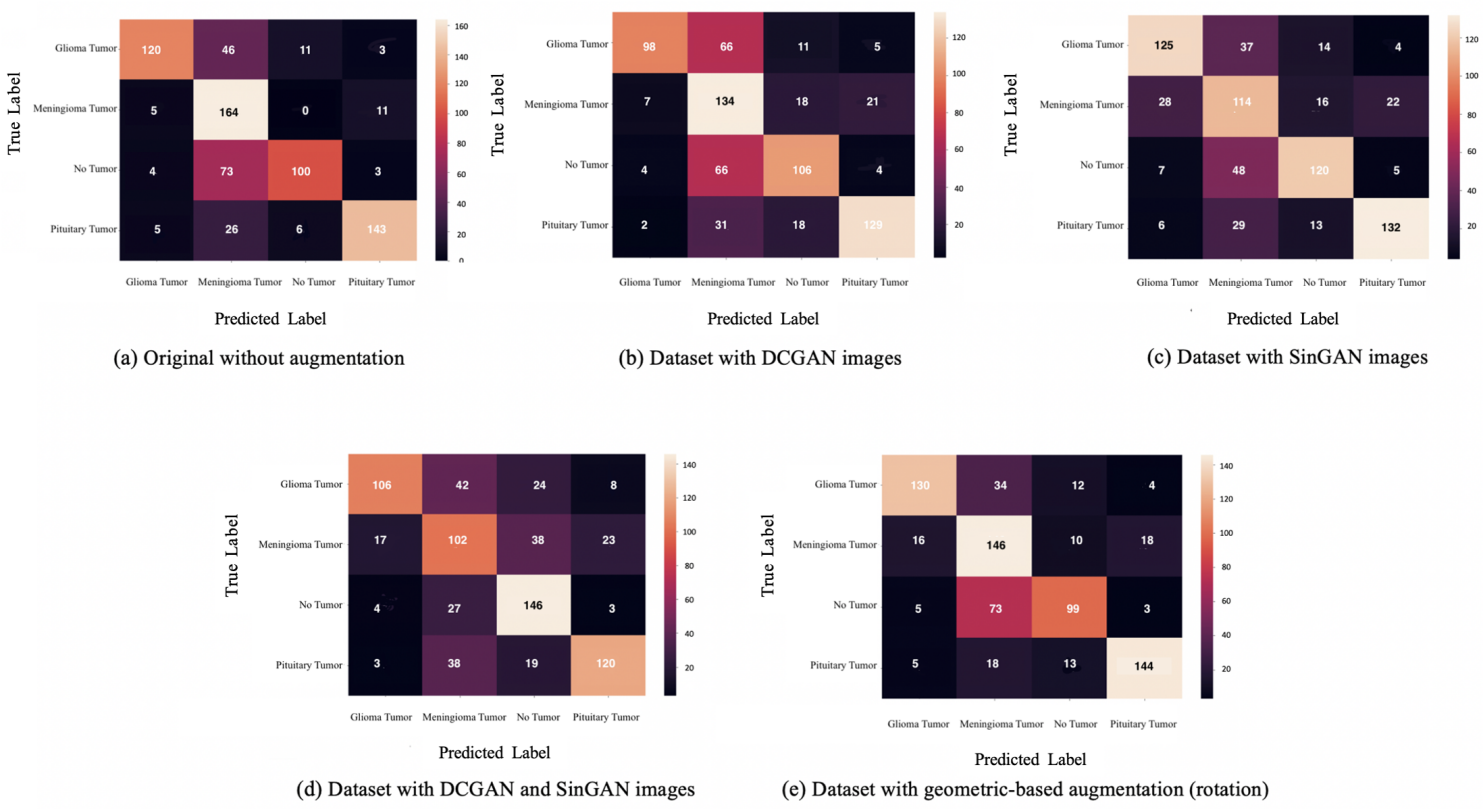
The confusion matrix for each dataset is presented here. Classifier bias towards the meningioma tumor type are shown in A-C. However, confusion matrices in (C) and (D) show a reduction in such bias after updating the dataset with SinGAN augmented images.

After augmentation, a VGG16 classifier was used (see model details in Table 9 and classifier parameters in Table 10). Besides, five versions of the dataset were used for classifications, (i) original dataset without augmentation, (ii) dataset with DCGAN augmented images, (iii) dataset with SinGAN augmented images, (iv) dataset with both DCGAN and SinGAN augmented images, and (v) dataset with geometric-based traditional augmentation. All five datasets have been equally split to 70% for training, 10% for validation, and 20% for testing. Note that 79 images from the original dataset in Table 2 were discarded to ensure the balance between the four classes, *i.e.,* each class had a total of 900 images and the total “no tumor” images before augmentation was 485 images. Table 11 describes the classification performance for each dataset. Interestingly, the original dataset without augmentation recorded the highest accuracy and largest loss. However, the dataset with SinGAN augmentation recorded the lowest loss. Moreover, Fig. 8 presents the training and validation loss for classifying each dataset. The augmentation techniques reduced the gap between the training and validation loss. Figure 9 shows the confusion matrices for each dataset and indicates the model’s bias towards the “meningioma tumor” class in all dataset versions, whereas the datasets that included SinGAN images reduced the classifier’s bias.

## Discussion

Regarding the results, brain tumor classification is not an easy task due to the similarities of brain tumor MRIs. The original brain MRIs dataset consists of imbalance classes, *i.e.,* small amounts of “no tumor” images compared with tumor images. Thus, the classifier recorded the highest testing loss after being trained with the original dataset (see Table 7). However, even though the authors implemented different augmentation techniques to solve the imbalance classification issue, the classification accuracy decreased in the augmented image dataset. The nature of brain MR images having precise details that differentiate between healthy and abnormal brain scans may have affected the generator’s performance. Thus, the generator generated images that negatively affected the classification model performance.

The geometric-based rotation technique, DCGAN, and SinGAN were used to augment “no tumor” images. Geometric augmentation techniques do not affect the inner parts of the brain image. However, augmenting brain MRIs with GAN-based techniques is critical; augmentation may affect the main brain conditions that indicate abnormal cases. DCGAN was trained with multiple images to produce fake “no tumor” MRIs. However, DCGAN performance was poor in terms of training duration and the generated images’ quality with high FID score (see Table 8). In addition, the SinGAN model trains on a single image, thus the augmented images look so real, *i.e.,* lower FID score compared to DCGAN.

**Table 8:** DCGAN and SinGAN FID scores.

Augmentation technique	Comparison case	FID score
DCGAN	“no tumor” generated images *vs.* original “no tumor” images	2.475
SinGAN	“no tumor” generated images *vs.* original “no tumor” images	1.292

**Table 9:** VGG16 model details.

Layer (type)	Output shape	Param #
Conv2d-1	[None, 64, 150, 150]	1,792
ReLU-2	[None, 64, 150, 150]	0
Conv2d-3	[None, 64, 150, 150]	36,928
ReLU-4	[None, 64, 150, 150]	0
MaxPool2d-5	[None, 64, 75, 75]	0
Conv2d-6	[None, 128, 75, 75]	73,856
ReLU-7	[None, 128, 75, 75]	0
Conv2d-8	[None, 128, 75, 75]	147,584
ReLU-9	[None, 128, 75, 75]	0
MaxPool2d-10	[None, 128, 37, 37]	0
Conv2d-11	[None, 256, 37, 37]	295,168
ReLU-12	[None, 256, 37, 37]	0
Conv2d-13	[None, 256, 37, 37]	590,080
ReLU-14	[None, 256, 37, 37]	0
Conv2d-15	[None, 256, 37, 37]	590,080
ReLU-16	[None, 256, 37, 37]	0
MaxPool2d-17	[None, 256, 18, 18]	0
Conv2d-18	[None, 512, 18, 18]	1,180,160
ReLU-19	[None, 512, 18, 18]	0
Conv2d-20	[None, 512, 18, 18]	2,359,808
ReLU-21	[None, 512, 18, 18]	0
Conv2d-22	[None, 512, 18, 18]	2,359,808
ReLU-23	[None, 512, 18, 18]	0
MaxPool2d-24	[None, 512, 9, 9]	0
Conv2d-25	[None, 512, 9, 9]	1,180,160
ReLU-26	[None, 512, 9, 9]	0
Conv2d-27	[None, 512, 9, 9]	2,359,808
ReLU-28	[None, 512, 9, 9]	0
Conv2d-29	[None, 512, 9, 9]	2,359,808
ReLU-30	[None, 512, 9, 9]	0
MaxPool2d-31	[None, 512, 4, 4]	0
AdaptiveAvgPool2d-32	[None, 512, 7, 7]	0
Linear-33	[None, 4096]	102,764,544
ReLU-34	[None, 4096]	0
Dropout-35	[None, 4096]	0
Linear-36	[None, 4096]	16,781,312
ReLU-37	[None, 4096]	0
Dropout-38	[None, 4096]	0
Linear-39	[None, 4]	16,388
Total params: 134,276,932		
Trainable params: 119,562,244		
Non-trainable params: 14,714,688		

**Table 10:** VGG16 classifier parameters.

Epochs	Learning rate	Batch size	Loss function	Optimizer
20	0.0001	128	Cross Entropy	Adam

**Table 11:** VGG16 classification performance.

Dataset	Number of images				Accuracy	Loss	Sensitivity	Precision
	Train	Validate	Test	Total				
Original without augmentation	2105	360	720	3185	**73**%	1.28	**73.19**%	**79.37**%
Dataset with DCGAN images	2520 (Synthetic = 415)	360	720	3600	64%	1.03	64.86%	70.96%
Dataset with SinGAN images	2520 (Synthetic = 415)	360	720	3600	68%	**0.85**	68.19%	69.98%
Dataset with DCGAN and SinGAN images	2520 (Synthetic = 415)	360	720	3600	65%	0.89	65.83%	68.15%
Dataset with geometric-based augmentation (rotation)	2520 (Synthetic = 415)	360	720	3600	71%	0.94	71.10%	74.08%

**Notes.**

* The bold text indicates the best results.

Moreover, the classifier was biased towards the “meningioma tumor” class in all datasets (see Fig. 9). This bias may be due to the similarities between brain tumor images. On the other hand, using SinGAN generated images reduced the classifier’s bias (see Figs. 9C and 9D). Additionally, the augmented dataset with SinGAN images reported the lowest loss compared with other augmented datasets (see Table 7).

According to the results, the geometric-based and SinGAN augmentation techniques are suitable to generate brain MRIs. However, these augmentation techniques need some improvements before adopting them. For instance, using different geometric-based augmentation techniques along with rotation, applying segmentation techniques with augmentation, fine-tuning the parameters, and optimizing the models. Moreover, due to the fine details that distinguish healthy from abnormal brain MRIs, GAN-based models and the classification performance were negatively affected. Therefore, brain MRIs require strong augmentation techniques that learn fine brain details.

## Conclusion

This effort aims to solve the imbalance issue in brain tumors dataset classes by enlarging the size of the class with small amounts of images. In this study, the selected dataset had a small “no tumor” class size, which needs to be increased. Thus, several experiments were conducted to generate the “no tumor” synthetic images using GAN-based and traditional-based augmentation techniques, *i.e.,* DCGAN, SinGAN, and rotation. However, the original dataset without any augmentation outperforms the other experiments in terms of accuracy, *i.e.,* 73%, while it obtained the highest loss. The highest loss explains the imbalance issue in the original dataset. Moreover, the traditional-based augmentation dataset achieved the second-best accuracy by 71%. Regarding the GAN-based images, SinGAN achieved the best accuracy by 68% and the lowest loss by 0.8525. However, the results illustrate that the healthy and abnormal brain MRIs are similar to each other (above 0.6 structural-based similarity scores). Moreover, the GAN-based models’ lack of attention to the fine details that distinguish healthy from abnormal brain MRIs. Thus, the nature of brain images causes a negative effect on classification performance. Consequently, the accuracy obtained in all the experiments needs to be improved, and the dataset size can be increased by augmenting all the dataset’s classes.

The authors plan to improve the generated fake images using different methods in future work, such as combining the data augmentation technique with reinforcement learning. Also, the authors will investigate different geometric-based augmentation techniques, such as flipping, zooming in and out, and other GAN types. Additionally, the classification and augmentation parameters can be changed to obtain optimal performance. The authors highly encourage interested researchers to explore the augmentation effect of sensitive medical images and enhance the available techniques accordingly.

## Additional Information and Declarations

### Data Availability

The following information was supplied regarding data availability: The data and code are available at GitHub: https://github.com/saraalrumih/Brain_tumor_augmentation_and_classification. The Brain Tumor Classification (MRI) dataset is available at Kaggle and Zenodo: https://www.kaggle.com/sartajbhuvaji/brain-tumor-classification-mri; Sarah S. Alrumiah, Norah Alrebdi, & Dina M. Ibrahim. (2023). Brain tumor augmentation and classification using GAN techniques. Zenodo. https://doi.org/10.5281/zenodo.7512471.
